# Post-translational regulation of P2X receptor channels: modulation by phospholipids

**DOI:** 10.3389/fncel.2013.00226

**Published:** 2013-11-25

**Authors:** Louis-Philippe Bernier, Ariel R. Ase, Philippe Séguéla

**Affiliations:** ^1^Department of Psychiatry, Brain Research Centre, University of British ColumbiaVancouver, BC, Canada; ^2^Department of Neurology and Neurosurgery, Alan Edwards Centre for Research on Pain, Montreal Neurological Institute, McGill UniversityMontréal, QC, Canada

**Keywords:** P2X receptors, ligand-gated channel, purine nucleotides, PIP2, phospholipids, pain, calcium, phospholipases

## Abstract

P2X receptor channels mediate fast excitatory signaling by ATP and play major roles in sensory transduction, neuro-immune communication and inflammatory response. P2X receptors constitute a gene family of calcium-permeable ATP-gated cation channels therefore the regulation of P2X signaling is critical for both membrane potential and intracellular calcium homeostasis. Phosphoinositides (PIP_n_) are anionic signaling phospholipids that act as functional regulators of many types of ion channels. Direct PIP_n_ binding was demonstrated for several ligand- or voltage-gated ion channels, however no generic motif emerged to accurately predict lipid-protein binding sites. This review presents what is currently known about the modulation of the different P2X subtypes by phospholipids and about critical determinants underlying their sensitivity to PIP_n_ levels in the plasma membrane. All functional mammalian P2X subtypes tested, with the notable exception of P2X5, have been shown to be positively modulated by PIP_n_, i.e., homomeric P2X1, P2X2, P2X3, P2X4, and P2X7, as well as heteromeric P2X1/5 and P2X2/3 receptors. Based on various results reported on the aforementioned subtypes including mutagenesis of the prototypical PIP_n_-sensitive P2X4 and PIP_n_-insensitive P2X5 receptor subtypes, an increasing amount of functional, biochemical and structural evidence converges on the modulatory role of a short polybasic domain located in the proximal C-terminus of P2X subunits. This linear motif, semi-conserved in the P2X family, seems necessary and sufficient for encoding direct modulation of ATP-gated channels by PIP_n_. Furthermore, the physiological impact of the regulation of ionotropic purinergic responses by phospholipids on pain pathways was recently revealed in the context of native crosstalks between phospholipase C (PLC)-linked metabotropic receptors and P2X receptor channels in dorsal root ganglion sensory neurons and microglia.

P2X receptor channels are involved in a wide variety of physiological processes ranging from sensory transduction to neuro-immune interactions to synaptic modulation. Upon binding to their agonist ATP, conformational changes induce the opening of a non-selective cation channel, impacting cellular physiology through membrane depolarization and calcium influx (North, 2002). This process is tightly controlled by various allosteric regulatory mechanisms, some acting on extracellular or transmembrane regions of the channel subunits, as is the case for metals, pH, divalent cations or alcohols (Coddou et al., 2011). On the other hand, several mechanisms of posttranslational regulation are known to affect the efficacy of P2X activation by interacting with the intracellular N- or C-terminal tails of the subunits. Among them, plasma membrane-bound lipids such as phosphoinositides (PIP_n_) were shown to have an important functional impact on P2X receptors, emerging as cofactors necessary for full channel activity. Here, we will review the recent evidence describing PIP_n_-dependent functional modulation for various members of the P2X receptor channel family, the molecular determinants of the protein-lipid interaction as well as the impact of this novel regulatory mechanism at the cellular level.

## Functional interactions between PIP_n_ and ion channels

PIP_n_ are composed of two fatty acid chains esterified to a glycerol backbone, attached to a *myo*-inositol ring forming a polar head group. Poly-PIP_n_ are synthesized through phosphorylations by selective PI kinases and are found in low abundance in cellular membranes, with phosphatidylinositol 4,5-bisphosphate (PI(4,5)P_2_; PIP_2_) being the most common, but only representing 1% of total cellular acidic lipids (Toker, 1998; Prestwich, 2004). PIP_n_ are classically viewed as critical players in ubiquitous intracellular signaling pathways. Notably, activation of phospholipase C (PLC) catalyses the hydrolysis of PIP_2_, giving rise to inositol trisphosphate (IP_3_) and diacylglycerol (DAG), triggering multiple signaling cascades (Berridge, 1993; Monserrate and York, 2010). The different species of PIP_n_ can also serve as membrane-bound anchors to various proteins, acting as a localization tag to specific organelles (Doughman et al., 2003; Heo et al., 2006). A third major signaling role of membrane PIP_n_ involves their direct functional regulation of integral membrane proteins (Suh and Hille, 2005; Gamper and Shapiro, 2007; Logothetis et al., 2010). Several families of channels and transporters have been demonstrated to be sensitive to PIP_n_ levels, among them are transient receptor potential (TRP) channels (Rohacs, 2007), inward-rectifier potassium channels (Kir) (Huang et al., 1998; Logothetis et al., 2007), KCNQ voltage-gated potassium channels (Hernandez et al., 2008b), cyclic nucleotide-gated (CNG) channels (Womack et al., 2000), epithelial sodium channels (ENaC; Kunzelmann et al., 2005), calcium release-activated calcium (CRAC) channels (Korzeniowski et al., 2009) and P2X receptor channels. Whereas direct protein-lipid binding was demonstrated for some of these families including P2X receptors, no consensus amino acid sequence has been defined to predict PIP_n_ binding to channels and transporters. However, several lines of evidence indicate that positive residues located on intracellular portions of the protein electrostatically interact with the negative head group of PIP_n_ to mediate the functional interaction (Rosenhouse-Dantsker and Logothetis, 2007; Whorton and Mackinnon, 2011).

## Functional regulation of P2X receptors by PIP_n_

### P2X1

The initial observation of PIP_n_-dependence for the P2X1 receptor subtype came from inside-out macropatch recordings in *Xenopus* oocytes expressing the receptor. Under this experimental condition, sequestration of PI(4,5)P_2_ by application of polylysine led to a current rundown, which could further be rescued by the addition of a soluble PI(4,5)P_2_ analog to the intracellular side of the membrane (Zhao et al., 2007). The regulatory role of PIP_n_ on P2X1 channel function was later confirmed when it was shown that blocking PI4 kinase (PI4K) activity negatively modulated P2X1 current amplitude and recovery from desensitization in whole-cell recordings performed on *Xenopus* oocytes expressing P2X1 (Bernier et al., 2008b). Current activation and desensitization rates were also slowed by PI(4,5)P_2_ depletion, suggesting a modulatory effect of the lipids on channel gating kinetics. However, PI3 kinase (PI3K) blockade did not affect P2X1 responses, indicating a prevalent role of PI(4,5)P_2_, as confirmed by the results showing a complete recovery of current kinetics and amplitude upon intracellular application of a soluble PI(4,5)P_2_ analog in the recorded oocyte following pharmacological depletion. A direct interaction between the proximal C-terminal region of P2X1 and various PIP_n_ was shown by the binding of fusion proteins containing the region of interest to phospholipid species including PI(4,5)P_2_ coated on nitrocellulose membranes (PIP strips). Mutating intracellular C-terminal basic residues into neutral glutamine (K359Q, K364Q) decreased the interaction affinity in this *in vitro* assay; P2X1 receptor containing these point mutations also exhibited decreased currents (Bernier et al., 2008b, 2012b). Interestingly, it was later shown that neutralizing lysine 364, as well as the positive arginine on position 360 also slowed the receptor recovery time after desensitization (Allsopp et al., 2013) in a mechanism that might implicate a decrease in PIP_n_ binding affinity.

### P2X1/5

The sensitivity of the P2X1/5 heteromeric subtype to phospholipids was studied in recombinant form via tranfection in HEK293 cells as well as in native expression in murine astrocytes acutely isolated from brain slices. In both preparations, the current carried through the P2X1/5 channel was decreased following PIP_n_ depletion. Under whole-cell patch-clamp configuration, the decreased channel responses could be rescued by addition of a PI(4,5)P_2_ analog inside the recording patch pipette. The direct positive regulation by the phospholipid, combined with the noticeable insensitivity of P2X5 homomers to PIP_n_ levels, is indicative of a critical dominant role of the P2X1 subunit in the regulation process (Ase et al., 2010). The extent to which calcium-permeable and PI(4,5)P_2_-sensitive P2X1/5 ATP-gated channels contribute to glial function and synaptic transmission still remains to be explored.

### P2X2

The first report of PIP_n_-dependence of a P2X receptor channel came from Fujiwara and Kubo, who demonstrated that P2X2 channel gating was affected by pharmacological depletion of PIP_n_ with the PI3K blockers wortmannin and LY294002 (Fujiwara and Kubo, 2006). They observed that the relative absence of PIP_n_ accelerated current desensitization, an effect mimicked by mutating two positively charged lysine residues of the proximal C-terminal region (K365 and K369) into neutral glutamines, indicating that an interaction between these two residues and PIP_n_ promotes the stability of the open conformation of the channel. Some P2X subtypes including P2X2 display unique activity- and time-dependent changes in channel permeability. By measuring the permeability shift in N-methyl-D-glucamine (NMDG^+^)-containing solutions, it was shown that this apparent pore dilation is also regulated by PIP_n_. The direct nature of the interaction between the C-terminus of P2X2 and PIP_n_ was demonstrated in two ways. By generating fusion proteins containing a region of interest from the C-terminal tail and applying them to PIP strips, it was confirmed that a direct binding can occur with several PIP_n_, including 3′ phosphorylated species dependent on PI3K activity. Using similar fusion proteins coupled to EGFP, the authors also reported association of the P2X2 proximal C-terminal tail to membrane PIP_n_ in COS-7 cells. Although these data lead to a primary role of PI(3)P and PI(3,5)P_2_, inside-out macropatch recordings performed by the Logothetis group showed that the application of PI(4,5)P_2_ can also directly rescue the rundown of P2X2 current induced by addition of polylysine, which binds and sequesters endogenous PI(4,5)P_2_ (Zhao et al., 2007).

### P2X3

As it was observed for all P2X homomers tested, the current rundown of the sensory P2X3 subtype expressed in *Xenopus* oocytes can be rescued by direct application of PI(4,5)P_2_ to its intracellular domains (Zhao et al., 2007). Mo et al. (2009) then provided evidence of the PI(4,5)P_2_-dependent regulation of the channel in native conditions, as ATP-evoked P2X3-mediated currents in dorsal root ganglion neurons were significantly decreased after PI4K inhibition with the furanosteroid wortmannin at micromolar concentrations (Mo et al., 2009). However, the interaction between P2X3 and PIP_n_ might involve indirect modulation, as no direct binding was found between various P2X3 C-terminal regions and PIP_n_ on PIP Strips. Furthermore, when expressed in heterologous systems like HEK293 cells or *Xenopus* oocytes, only the rate of recovery from desensitization of the receptor was affected by PI(4,5)P_2_ levels. The absence of direct PIP_n_ binding *in vitro* and the striking cell type-dependent difference in functional regulation strongly suggests that an unidentified associated protein expressed in DRG neurons provides a necessary link between P2X3 channels and phospholipids.

### P2X2/3

The currents carried through the P2X2/3 heteromer were also shown to be modulated by pharmacological PIP_n_ depletion in *Xenopus* oocyte expression system and in native conditions in rat dorsal root ganglion neurons (Mo et al., 2009). Functionally, the P2X2/3 receptor channel retains the PIP_n_ sensitivity of the two subunits found in the heteromer. Blocking the formation of 3′ phosphorylated PIP_n_ with wortmannin reduced its current amplitude, as is the case for the P2X2 homomer. On the other hand, the blockade of the PI4K-dependent synthesis of 4′ phosphorylated isoforms also inhibited currents, similar to what is seen for the P2X3 homomer. Moreover, following PIP_n_ depletion, the P2X2/3 current amplitude can be partially rescued by addition of either PI(3,4,5)P_3_ or PI(4,5)P_2_.

### P2X4

The PIP_n_ sensitivity of the P2X4 receptor channel has been extensively studied in both recombinant systems and native models. In inside-out macropatch, PI(4,5)P_2_ increases currents carried through the P2X4 channel (Zhao et al., 2007), while whole-cell currents are inhibited by depletion of PI(3,4,5)P_3_ or PI(4,5)P_2_ (Bernier et al., 2008a). Subsequent intracellular injection of either of these two major signaling phospholipids leads to a recovery of the P2X4 current. Following PIP_n_ depletion, P2X4 also exhibits a slower recovery from desensitization as well as slower activation and desensitization rates, suggesting that PIP_n_ binding to the channel subunit triggers a conformational change that affects its gating, as was also observed with P2X1 and P2X2. P2X4-mediated ATP responses in BV2 murine microglial cells were recorded in patch clamp, and there again, PIP_n_ act as cofactors necessary for full current amplitude. Since the P2X4 channel has the highest calcium permeability in the P2X family (Egan and Khakh, 2004), P2X4-mediated calcium entry in microglia was also assayed and a similar inhibition following PIP_n_ depletion was observed. The capability of P2X4 to form a large conductance pore upon sustained activation was also found to be dependent on PIP_n_ when tested in a YO-PRO-1 uptake assay in primary mouse microglia and in an assay measuring time-dependent changes in NMDG^+^ permeability of the channel in the *Xenopus* oocyte heterologous expression system (Bernier et al., 2012a). Binding between a proximal region of the P2X4 C-terminal tail and various PIP_n_ was demonstrated using PIP strips, and individual basic residues (K362, K363, K370, K371) were found to be critical for high-affinity lipid-protein (Bernier et al., 2008a, 2012b). In functional assays, mutating two of these residues to glutamine, therefore neutralizing their positive charge and decreasing the affinity of the P2X4-PIP_n_ binding, induced a current that showed a slower recovery from desensitization, a slower activation rate and a slower desensitization rate. To further confirm the ability of the P2X4 proximal C-terminal region (C360-V375) to bind to membrane PIP_n_ in cellular environments, a whole-cell patch-clamp experiment was performed in P2X4-expressing HEK293 cells, where fusion proteins containing the putative PIP_n_-interacting peptide of P2X4 were introduced in the patch pipette. The addition of the P2X4 C360-V375 peptide led to a decrease in the activation and desensitization rates of the P2X4 channel, indicating a competition for PIP_n_ binding between the P2X4 receptor and its own PIP_n_-binding domain. Conversely, introducing fusion proteins containing PIP_n_ binding loss-of-function mutations abrogated the effect. Overall, these results suggest that P2X4 binds directly to PI(3,4,5)P_3_ and PI(4,5)P_2_ via key lysine residues in the proximal C-terminal region, and that this interaction leads to conformational changes increasing the efficacy of channel activation.

### P2X5

Functional data obtained in heterologous expression systems suggest that the P2X5 homomer is the only functional mammalian P2X subtype insensitive to PIP_n_ levels in the plasma membrane. A first report showed that when expressed in HEK293 cells, P2X5 was unaffected by pharmacological depletion of PIP_n_ (Ase et al., 2010). Expectedly, no direct binding between the P2X5 C-terminal tail and PIP_n_ could be observed using PIP strips. When various mutations were introduced on the P2X5 C-terminus to improve its binding affinity to PIP_n_, the current carried by the P2X5 channel was greatly increased, and the mutated receptor acquired sensitivity to wortmannin-induced PIP_n_ depletion, implying that the relatively small currents obtained in WT P2X5 were due to a lack of PIP_n_-dependent potentiation (Bernier et al., 2012b). Furthermore, the PIP_n_-binding mutant P2X5 exhibited a faster recovery from desensitization, as well as faster activation and desensitization rates, whereas pharmacological PIP_n_ depletion led to a current phenotype similar to that observed with wild-type P2X5. The profound changes seen in the gating properties of the PIP_n_-binding P2X5 mutant further confirms the important functional role phospholipids play on P2X function.

### P2X7

The initial observation of PIP_n_-dependence of the P2X7 receptor came from the Logothetis group, who demonstrated that pharmacological inhibition of PI(4,5)P_2_ synthesis reduced the P2X7 current density in *Xenopus* oocytes and HEK293 cells (Zhao et al., 2007). PLC-dependent PI(4,5)P_2_ hydrolysis induced by platelet-derived growth factor receptor (PDGFR) activation was also shown to partially inhibit P2X7 function and addition of PI(4,5)P_2_ directly reversed the rundown of P2X7 current in inside-out macropatches. Furthermore, three positively-charged amino acid residues of the C-terminal domain were found to be critical in the PI(4,5)P_2_-dependence of the P2X7 receptor. However, no direct binding was observed between the P2X7 C-terminal tail and PIP_n_ on PIP strip membranes, suggesting that the channel-lipid interactions might be indirect (Bernier et al., 2012b).

## Molecular determinants of the PIP_n_ interaction with P2X receptors

**Figure 1:**
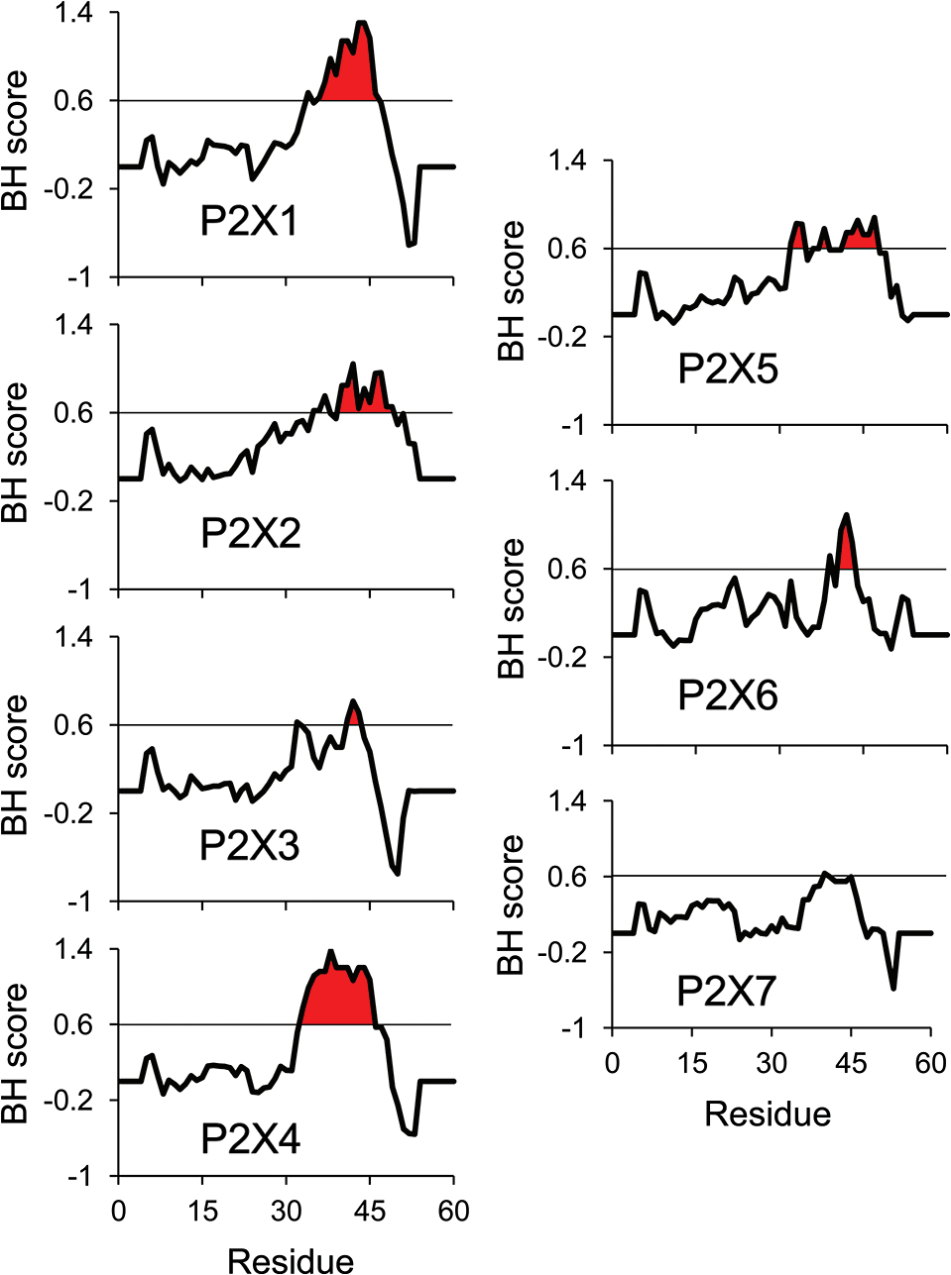
**BH scale analysis of P2X C-terminal regions.** The analysis of all rat P2X C-terminal regions using the BH scoring method (Brzeska et al., 2010) predicts strong PIP_n_ affinity for the reported PIP_n_ binding region in the subunits experimentally shown to directly bind PIP_n_. A BH score above 0.6 was demonstrated to accurately identify unstructured lipid-binding sites in proteins. The input sequence consisted of 60 amino acids of the C-terminal region, including the last 13 amino acids of the second transmembrane domain (starting at residue Gly324 in P2X1 numbering). The analysis was performed with a window size of eight amino acids.

As all ATP-activated P2X subtypes except P2X5 were shown to be functionally regulated by PIP_n_, it is critical to characterize the nature of the protein-lipid interaction. For all PIP_n_-sensitive subtypes, several basic amino acid residues were shown through mutational assays to be necessary for the regulation. This is in accordance with most PIP_n_-binding regions of other types of proteins; while no consensus sequence exists for PIP_n_ binding, the presence of positively-charged residues, often found in clusters, is necessary for an electrostatic interaction to take place with the negatively-charged head of the lipid (Rosenhouse-Dantsker and Logothetis, 2007; Whorton and Mackinnon, 2011). For P2X receptors, it is hypothesized that an intracellular domain containing a dual cluster of basic amino acids, mainly arginines and lysines located on the C-terminal tail 6 to 19 residues away from the second transmembrane domain, is required for PIP_n_ binding (Bernier et al., 2012b). Some subtypes, including P2X3, P2X7 and the PIP_n_-insensitive P2X5 lack this characteristic domain and hence do not directly bind to PIP_n_. Analysis of the basic and hydrophobic (BH) score of P2X C-terminal regions, using a quantification method that measures the lipid binding affinity of unstructured linear protein sequences (Brzeska et al., 2010), also predicts the presence of a lipid binding site on most P2X subunits (Figure 1). The high BH score region corresponds to the dual polybasic cluster motif experimentally confirmed (Bernier et al., 2012b). Furthermore, mutational analysis of P2X5 indicated that the regulatory dual cluster motif is not only necessary, but also sufficient for PIP_n_-dependent regulation, as creation of the putative PIP_n_ binding site via neutral- or acidic-to-basic mutation induced a current phenotype functionally modulated by PIP_n_. Similar regulatory PIP_n_-binding motifs have been found on multiple PIP_n_-dependent channels, including members of the TRPM family which bind to PIP_n_ via the TRP box, and M-type potassium channels (Kv7) interacting with PIP_n_ via a polybasic cluster (Rohacs et al., 2005; Nilius et al., 2008; Hernandez et al., 2008a; Hansen et al., 2011).

Whereas direct binding between phospholipids and the binding motif *in vitro* correctly predicts sensitivity to PIP_n_ and phenotype for most P2X subtypes, results obtained with P2X3 and P2X7 indicate that indirect PIP_n_ modulation can also influence channel properties. Several acidic residues are found within the PIP_n_ binding site of P2X3, disrupting the global positive charge of the clusters and reducing its binding affinity. On the other hand, the P2X7 sequence displays only one polybasic cluster. Accordingly, no direct binding to PIP_n_ was observed for the two subtypes (Mo et al., 2009; Bernier et al., 2012b). However, functional regulation of the P2X3 and P2X7 channels by PIP_n_ was observed in various models, leading to the hypothesis of an association with a cofactor acting as an indirect sensor of PIP_n_ levels in the plasma membrane (Zhao et al., 2007; Mo et al., 2009). Indirect interactions have been demonstrated for various PIP_n_-sensitive channels: the potentiation of TRPV1 by PIP_n_ relies on *p*hosphoinositide *i*nteracting *r*egulator of *T*RP (PIRT) acting as a linker between both molecules, and N-methyl-D-aspartate (NMDA) glutamate receptors are regulated by PIP_n_ via α-actinin interacting with both the lipids and the NR1 and NR2b subunits to promote channel opening (Michailidis et al., 2007; Kim et al., 2008). Interestingly, the long P2X7 C-terminal domain directly associates with various proteins including α-actinin, possibly linking the channel subunit to PIP_n_ (Kim et al., 2001).

**Figure 2:**
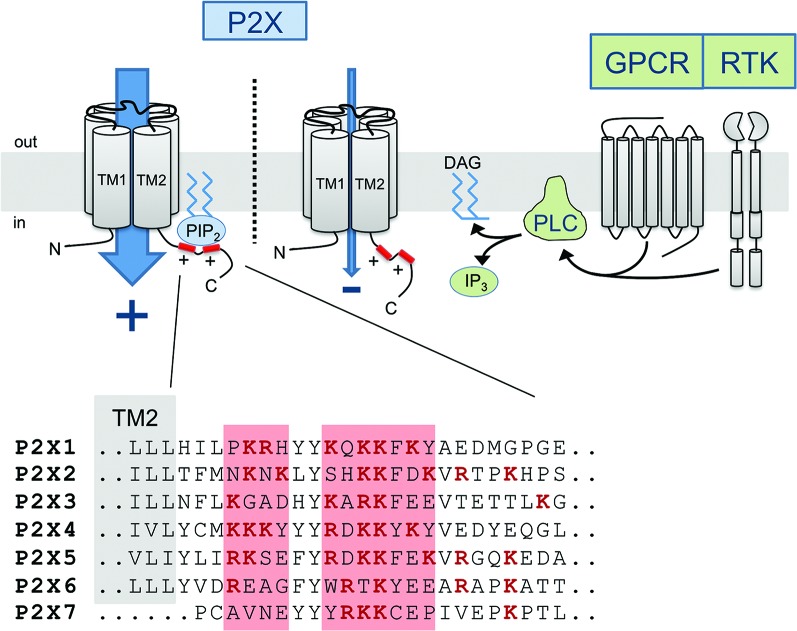
**Diagram of the PIP_n_-dependent metabotropic regulation of P2X receptor channels.** Membrane-bound PIP_n_ directly bind a dual polybasic cluster motif found in the C-terminal region of certain P2X receptor subtypes, modulating the current carried through the channel. G protein-coupled receptor (GPCR) or receptor tyrosine kinase (RTK) activation induces PLC-mediated hydrolysis of PI(4,5)P_2_, transiently reducing the levels of PI(4,5)P_2_ and affecting P2X function. The amino acid sequence of the proximal C-terminal regions of P2X receptors shows the presence of two clusters of basic residues forming a regulatory PIP_n_ binding site in most subunits.

The exact molecular mechanism by which protein-PIP_n_ binding induces changes in the functional phenotype of P2X channels remains elusive. However, it is likely that such interaction, whether it be direct or indirect, triggers a conformational change in TM2 linked to the proximal C-terminal domain. Recent evidence from crystallization of the PIP_n_-binding and PIP_n_-sensitive Kir2.2 channel in the presence of PIP_2_ demonstrates that a similar channel-lipid interaction can lead to significant movements of the cytosolic domains, by as much as 6 Å (Haider et al., 2007; Hansen et al., 2011; Whorton and Mackinnon, 2011). The recent crystallographic data of P2X4 unfortunately do not contain the cytosolic C-terminal domain where the P2X PIP_n_ binding site is located (Kawate et al., 2009; Hattori and Gouaux, 2012). Interestingly, the regulatory PIP_n_ binding site, being in close proximity to the second transmembrane domain, is located only 15 to 20 amino acids away from the gate region of the pore controlling the ion conduction properties of the channel (Figure 2). Since conformational changes in the gate area upon ATP binding mediate the opening of the channel as well as its desensitization (Kracun et al., 2010; Li et al., 2010; Kawate et al., 2011; Du et al., 2012; Hattori and Gouaux, 2012), it is likely that forces generated by lipid binding affect the movements of the region and alter the gating kinetics of the channel, as PIP_n_ level-dependent changes in channel activation and desensitization rates have been observed in most P2X subtypes.

Furthermore, the ability of P2X2 and P2X4 receptor channels to dilate into a large conductance pore is also sensitive to PIP_n_ levels (Fujiwara and Kubo, 2006; Bernier et al., 2012a). This activity-dependent change in permeability is believed to be driven in part by major rearrangements in the interactions between the transmembrane domains (Eickhorst et al., 2002; Chaumont and Khakh, 2008; Shinozaki et al., 2009). For P2X2, 10 residues in the transmembrane domains were shown to be involved in the transition to the high permeability state, several of which are in close proximity to the PIP_n_ binding site (Fisher et al., 2004; Khakh and Egan, 2005). Also arguing for a role of PIP_n_ in regulating the large pore formation via conformational changes are reports demonstrating that the change in permeability requires rearrangements of the cytosolic domains where the channel-lipid interaction site is found. More specifically, a study using a chimeric P2X2 engineered with a PIP_2_-binding pleckstrin homology (PH) domain fused to its C-terminal tail showed that PIP_2_ can tether the domain to the membrane, thereby preventing the transition of P2X2 into a dilated state (Fisher et al., 2004).

While we can speculate that PIP_n_ binding induces a rearrangement of the C-terminal tail of P2X channels and in this way changes its functional properties, it remains unclear how specific the interaction is with regards to the various PIP_n_ species present in cellular environments. P2X1, P2X3 and P2X7 seem to be strongly potentiated by PI(4,5)P_2_, P2X2 is mainly modulated by the 3′ phosphorylated PIP_n_ [PI3P, PI(3,5)P_2_], while P2X4 depends on both PIP(4,5)P_2_ and PI(3,4,5)P_3_ as cofactors for full activation. Parallels will be drawn between P2X receptors and other families of PIP_n_-dependent ion channels in terms of protein-lipid binding characteristics. Most phospholipid-dependent channels and transporters require PI(4,5)P_2_ however some are modulated by PI3P and PI(3,5)P_2_, like small-conductance Ca^2+^-activated K^+^ channels, or by PI(3,4,5)P_3_, like CNG channels and epithelial sodium channels (Zhainazarov et al., 2004; Pochynyuk et al., 2005; Srivastava et al., 2006). For P2X receptor channels, while subtype-specific variations in the primary sequence of the regulatory PIP_n_ site could provide some binding specificity to PIP_n_ species, it is likely that the regulation is mostly directed by the relative abundance of specific PIP_n_ in the membrane microenvironment surrounding the channel. Unlike PH domains which require a complex protein folding and basic residues scattered over a region of hundreds of amino acids, the shorter and more linear P2X PIP_n_ binding site only forms a cluster of positive charges electrostatically interacting with the negative head group of the lipid, providing a lower level of specificity.

## A physiological role for PIP_n_ regulation of P2X

Intracellular PIP_n_ levels can fluctuate very rapidly within the plasma membrane. Many surface receptors are coupled to the activation of PLC isoforms, transiently lowering the levels of PI(4,5)P_2_ via hydrolysis, while enzymes involved in PIP_n_ synthesis, like PI3K or the phosphatase and tensin homolog (PTEN), are tightly regulated via multiple signaling cascades. Therefore, receptor channels requiring the presence of PIP_n_ as essential cofactor for complete function can be regulated by enzyme-driven depletion or addition of PIP_n_ species. Multiple examples of PIP_n_-dependent ion channel regulation through metabotropic pathways exist, including M1 muscarinic receptor-mediated inhibition of KCNQ channels, Trk- and PLC-mediated inhibition of TRPM7 or G protein-coupled inwardly-rectifying potassium (GIRK) channels (Caulfield et al., 1994; Kobrinsky et al., 2000; Runnels et al., 2002; Cho et al., 2005; Brown et al., 2007; Falkenburger et al., 2010). The first report of P2X receptor channels being regulated through receptor-initiated depletion of PI(4,5)P_2_ came from the Logothetis group, who showed that P2X7 currents are inhibited by co-activation of PDGFR in *Xenopus* oocytes (Zhao et al., 2007). The inhibition specifically depends on PIP_2_ hydrolysis as it does not occur following activation of a PLCγ-deficient mutant PDGFR. Another multireceptor crosstalk involving PIP_n_ was later reported natively as cationic currents carried through the P2X3 receptors in isolated dorsal root ganglion neurons are reduced after activation of the UTP-sensitive P2Y2 G_q_ protein-coupled receptor (Mo et al., 2013). This interaction can be occluded by exogenous introduction of a PIP_2_ analog as well as by pharmacologically uncoupling the P2Y2 receptor from PLC activation, indicating that P2X3 inhibition by P2Y2 directly relies on PI(4,5)P_2_ hydrolysis. A third crosstalk was recently uncovered in microglia, where P2X4 receptor channels are functionally inhibited by co-activation of the G_q_-coupled P2Y6 receptor also upregulated in neuropathic pain conditions (Bernier et al., 2013). UDP activation of P2Y6 leads to a PLC-dependent decrease in P2X4-mediated currents and calcium entry in both resting and LPS-activated microglia. The dilation of P2X4 into a large conductance pore was also inhibited by P2Y6 activation, all of these effects being highly similar to that of pharmacological depletion of PIP_n_.

These recent results suggest that the requirement of PIP_n_ as a cofactor for P2X receptor is a critical regulatory mechanism involved in signaling crosstalks under physiological and pathological conditions. Many aspects of this post-translational modulatory mechanism remain to be investigated to understand its physiological significance. For example, it is still unclear if and how this modulatory pathway can be specific to one metabotropic receptor. Can P2X receptors be inhibited by any signaling event inducing PLC hydrolysis of PI(4,5)P_2_? Interestingly, stimulating G_q_-coupled P2Y1 ADP receptors or M3 muscarinic receptors failed to inhibit P2X4 responses in transfected HEK293 cells while stimulating P2Y6 receptors did, indicating some degree of specificity to the P2Y6-P2X4 crosstalk (Bernier et al., 2013). It is probable that subcellular localization plays a key role in controlling which receptors interact. PI(4,5)P_2_ hydrolysis by PLC likely induces only a local depletion in PI(4,5)P_2_ levels and could therefore preferentially affect adjacent P2X receptors. It would be interesting to investigate the role of lipid rafts in controlling the proximity of different receptors, considering that plasma membrane microenvironments have been reported to affect P2X physiology (Vacca et al., 2004; Vial and Evans, 2005; Allsopp et al., 2010). Other factors such as GPCR desensitization might also come into play given that PIP_n_ levels have a fast turnover rate. For example, P2Y6 displays a much slower desensitization pattern than other P2Y receptors and might induce a longer, more significant decrease in membrane PI(4,5)P_2_ level (Robaye et al., 1997).

As an increasing amount of data highlights the role of PIP_n_ in P2X receptor activity and multireceptor crosstalks, such a post-translational regulatory mechanism might provide an innovative pharmacological target to treat chronic pain conditions or immune diseases where P2X receptors are known to be involved.

## Conflict of interest statement

The authors declare that the research was conducted in the absence of any commercial or financial relationships that could be construed as a potential conflict of interest.
